# Framing optional genetic testing in the context of mandatory newborn screening tests

**DOI:** 10.1186/s12911-015-0173-3

**Published:** 2015-06-27

**Authors:** Sarah E. Lillie, Beth A. Tarini, Nancy K. Janz, Brian J. Zikmund-Fisher

**Affiliations:** Center for Chronic Disease Outcomes Research, Minneapolis VA Health Care System, Minneapolis, USA; Child Health Evaluation and Research (CHEAR) Unit, Department of Pediatrics, University of Michigan, Ann Arbor, USA; Division of General Medicine, Department of Internal Medicine, University of Michigan, Ann Arbor, USA; Department of Health Behavior & Health Education, School of Public Health, University of Michigan, Ann Arbor, USA; Center for Bioethics and Social Sciences in Medicine, University of Michigan, Ann Arbor, USA; Risk Science Center, School of Public Health, University of Michigan, Ann Arbor, USA

**Keywords:** Newborn screening, Decision making, Duchenne muscular dystrophy, Optional newborn screening

## Abstract

**Background:**

Parents are increasingly faced with decisions about optional newborn bloodspot screening (NBS) despite no consistent policy for communicating information about such testing. We examined whether framing optional NBS alongside mandatory NBS influenced intention to participate in optional NBS.

**Methods:**

For this Internet-administered study, 2,991 adults read a hypothetical vignette in which optional NBS for Duchenne muscular dystrophy (DMD) was either presented by itself (in isolation), alongside a description including the total number of mandatory NBS tests (“bundled” mandatory context), or alongside a listing of each mandatory NBS test (“unbundled” mandatory context). We assessed associations with participants’ intended participation using ordered logistic regression models, and associations with attitudes towards optional DMD NBS and subjective norms using Analysis of Variance.

**Results:**

Participants were more likely to choose optional DMD NBS if they also read information about mandatory NBS (either bundled or unbundled) versus when DMD NBS was presented in isolation. Participants who read about optional DMD NBS in isolation also reported such testing to be less important and that they would worry more about the results than those who also saw mandatory NBS information.

**Conclusions:**

Future NBS programs should pay attention to the framing of optional testing communication, as it influences parental behavior. Predictors of NBS uptake will become increasingly important as NBS programs continue expanding.

**Electronic supplementary material:**

The online version of this article (doi:10.1186/s12911-015-0173-3) contains supplementary material, which is available to authorized users.

## Background

Newborn bloodspot screening (NBS) is a highly successful public health program aimed at the early identification of babies with potentially devastating conditions that benefit from pre-symptomatic diagnosis and treatment. In the United States alone, over 4 million infants per year are screened through state-based mandatory programs [1]. The number of disorders included in NBS programs has greatly increased over the last decade due to advances in testing technology [2, 3]. During this period the American College of Medical Genetics advocated for a uniform core NBS panel of 29 conditions, which most states adopted [4].

Since the release of the Recommended Uniform Screening Panel [5] there has been increasing uniformity in the types of disorders for which newborns are screened. However, states can still screen for conditions beyond the Recommended Uniform Screening Panel (e.g., New York State includes NBS for Krabbe Disease), leading to variation in NBS programs [6]. Adding further to this inconsistency is the existence of “pilot studies”, which offer optional NBS for diseases not included in mandatory NBS panels for which there is limited treatment efficacy data [7, 8]. Such pilot studies evaluate the efficacy and safety of adding a new test into mandatory NBS programs [8]. In addition to this inconsistency in the conditions NBS programs test for, there is inconsistency in the timing, methods, and sources of information that parents receive about NBS [9]. The expansion of NBS and variability in how NBS information is delivered, including what information is included and the way in which this information is presented, leads to questions about how optional NBS, increasingly available, is communicated.

Thus far, most research on optional NBS has focused on parental knowledge and attitudes, finding overwhelming parental support for NBS [10–17]. Although few studies have assessed attitudes towards information provision, addressing the timing and source of information [9, 18], no studies have explored the framing of optional NBS, specifically the mandatory NBS context in which optional NBS is presented. For example, several pilot programs have offered optional NBS for Duchenne muscular dystrophy (DMD), most recently the Centers for Disease Control-funded Statewide Newborn Screening for Duchenne Muscular Dystrophy at the Columbus Children’s Research Institute in Ohio [19]. DMD is a rare, lethal form of muscular dystrophy that does not fulfill the criteria for inclusion in mandatory NBS; there is no effective treatment that justifies earlier testing in the newborn period [20, 21]. Researchers have studied DMD NBS attitudes and recently begun to identify factors influencing the parental decision-making process in DMD NBS [17, 22–24], but the context of how DMD NBS information is presented has yet to be studied. DMD NBS could be presented by itself, which may encourage parents to consider it solely based on its intrinsic characteristics. Alternately, it could be presented with information about existing mandatory NBS programs, either generalized information or detailed. It is unclear whether framing optional NBS in this way would encourage its use by connecting parents to the pros of mandatory NBS, or discourage it by making optional NBS seem trivial or excessive, as yet another test on top of the many already being done.

To explore the question of whether contextual framing of optional NBS affects decision making, we conducted an experimental study in which we randomized whether an optional NBS test (DMD NBS) was presented by itself, in comparison with a description of mandatory NBS that simply described the total number of tests, or as part of a description of mandatory NBS that listed each mandatory test separately. We assessed the effect of these conditions on DMD NBS intention and attitudes about DMD NBS.

## Methods

### Study population

We recruited adult Internet users through Amazon’s Mechanical Turk (MTurk), an online marketplace for people willing to complete tasks (such as surveys) that cannot be automated [25]. Inclusion criteria included age 21 years or older, United States resident, and the ability to complete a web-based survey in English. Because MTurk users tend to be younger adults, we expected that this sampling strategy would yield mostly adults who were either relatively recent parents or potentially contemplating having children. Participants were paid $0.75 upon completion of the survey, which is consistent with prevailing rates on MTurk for surveys of this length. This study was declared exempt by the Institutional Review Board at the University of Michigan.

### Procedure

To begin each study, participants read a vignette that outlined a hypothetical situation in which they have a newborn son and are presented with basic information about optional DMD NBS. Participants then read a brochure that provided a detailed description of DMD and DMD NBS. Participants were randomized into one of the three experimental groups: isolation context, bundled mandatory NBS context, and unbundled mandatory NBS context. For those in the isolation context group, the brochure discussed only the optional NBS; mandatory NBS was not mentioned. Participants in the bundled mandatory NBS context group saw a brochure in which mandatory NBS was discussed, but only as an aggregated package (“50 tests”). Participants in the unbundled mandatory NBS context group saw a more detailed brochure in which each of the 50 mandatory NBS tests was listed separately. The purpose of the bundled and unbundled presentations was to frame DMD NBS in the context of mandatory NBS, either as an addition to a single package of tests (bundled) or as an addition to an exhaustive list of tests (unbundled). See Additional file 1 for the exact brochures shown to participants. The format of and basic information included in the brochures were modeled after the study brochure from the Statewide Newborn Screening for Duchenne Muscular Dystrophy at the Columbus Children’s Research Institute [19].

### Measures

The main outcome measure was intention to participate in DMD NBS. After reading the vignette and brochure, participants reported how likely it was that they would choose DMD NBS (4-point Likert scale; “Very Unlikely” to “Very Likely”). Due to the hypothetical nature of this study, we wanted to identify those participants who had a clear desire for NBS. Therefore for our analyses this variable was dichotomized into chose DMD NBS (score of 4) vs did not chose DMD NBS (score of 1–3).

Participants answered eight questions that assessed their attitudes about DMD NBS, drawn from previous qualitative work regarding parents’ responses to DMD NBS [13, 14]. First, participants rated the importance of…1) DMD NBS; 2) seeing the results of DMD NBS; and 3) sharing the results of DMD NBS (5-point Likert scales; “Not at all Important” to “Very Important”). Then participants responded to the following five questions/statements, each on a 5-point Likert scale: 1) How much do you think you would worry about the results of your baby’s DMD test? (“Not at all” to “Very Much”); 2) The information from the DMD test may help me prepare for the future (“Strongly Disagree” to “Strongly Agree”); 3) The information from the DMD test would affect whether I have more children (“Strongly Disagree” to “Strongly Agree”); 4) The results from the DMD test may affect how I treat my child (“Strongly Disagree” to “Strongly Agree”); 5) My child would be treated differently by others if he is diagnosed with DMD (“Strongly Disagree” to “Strongly Agree”).

Of course, decision making also occurs within a social context [26], and subjective norms have influenced both newborn [27] and prenatal [28] screening. Thus we assessed the influence of optional NBS framing on social normative beliefs around two topics: NBS in general and DMD NBS specifically. Participants answered two questions (7-point Likert scale; “Agree” to “Disagree”): 1) Do you think that most people agree or disagree that it is important for babies to be tested for as many genetic diseases as possible?; and 2) Do you think that most people agree or disagree with getting DMD NBS?. We expected that presenting DMD NBS within the context of the larger, mandatory NBS program would lead to stronger subjective norm attitudes.

At the end of the survey, participants reported demographic information, including age, race, gender, marital/partnered status, level of education, and household income. We assessed participants’ experiential knowledge of NBS, measured by either their or a partner’s pregnancy history.

### Analysis plan

To examine the influence of contextual framing on choosing optional DMD NBS, we conducted ordered logistic regression analyses (with and without demographic predictors) using the dichotomized intended participation variable. We also conducted ANOVA analyses to test the influence of contextual framing on attitudes about DMD NBS. Control factors included demographic variables and previous pregnancy. All analyses were completed using STATA [Stata Statistical Software: Release 12. College Station, TX: StataCorp LP].

## Results

### Study participants

A total of 3,215 participants completed surveys; 224 surveys were excluded for participants reporting an age less than 21 years per the exclusion criteria, resulting in a final N of 2,991. Participants were predominately white (79.9 %), male (52.4 %), and had no history of pregnancy (63.0 %). The minority (39.9 %) were married/partnered. The average age was 29.3 years (range 21–82, SD = 9.5; 81.1 % under 36 years). A large minority of participants (42.1 %) had a college degree, but 9.8 % had a high school education or less. See Table 1 for full participant characteristics.

**Table 1:** Participant characteristics (N = 2,991)^*a*^

Characteristic	% (N)
*Age (range, mean SD)*	21-82, 29.3 (9.5)
21-35	81.1 (2,099)
36 and older	18.9 (489)
*Race*	
White	79.9 (2,387)
African American	5.8 (172)
Asian	8.3 (247)
Other	6.0 (180)
*Gender*	
Male	52.4 (1,563)
Female	47.6 (1,419)
*Marital Status*	
Married/partnered	39.9 (1,189)
Not married/partnered	60.1 (1,791)
*Education Level*	
Some high school	1.1 (34)
High school/GED	8.7 (259)
Some college/Tech	37.1 (1,109)
College degree	42.1 (1,259)
Advanced degree	11.0 (328)
*Household Income*	
<$14.5k^*b*^	15.0 (448)
$14.5 to < $35 k	26.0 (776)
$35 k to < $50 k	19.3 (575)
$50 k to < $75 k	19.1 (570)
$75 k - < $100 k	11.1 (330)
$100 k and over	9.5 (282)
*Previous Pregnancy* ^*c*^	
Yes	36.0 (1,078)
No	63.0 (1,883)
Don’t Know	1.0 (30)

^*a*^N varies due to missing data. Percentage missing data < 0.50 % for all variables except age (13.41 % missing)

^*b*^k = thousand

^*c*^Own or partner’s pregnancy

### Influence of contextual framing on intended participation in DMD NBS

Participants given information about DMD NBS in a mandatory NBS context (either bundled or unbundled) were more likely to choose DMD NBS, compared to those given information without any reference to mandatory NBS (Bundled: OR = 1.43, CI=1.16, 1.75, *p* < 0.01; Unbundled: OR = 1.38, CI=1.13, 1.68, *p* < 0.01). Certain demographic characteristics predicted likelihood of choosing DMD NBS (Table 2). In particular, previous pregnancy, lower education attainment, and African American race all predicted lower intention to pursue optional DMD NBS.

**Table 2:** Predictors of choosing Duchenne muscular dystrophy (DMD) newborn screening (NBS) (*N* = 2,562)^*a*^

Variable	OR (95 % CI)
*Contextual Framing*	
Isolation	Reference
Bundled mandatory NBS	1.43 (1.16, 1.75)**
Unbundled mandatory NBS	1.38 (1.13, 1.68)**
*Age*	
21-35	Reference
36 and older	0.95 (0.75, 1.19)
*Race*	
White	Reference
African American	0.64 (0.45, 0.90)*
Asian	1.36 (0.98, 1.89)
Other	1.11 (0.79, 1.58)
*Gender*	
Male	Reference
Female	1.19 (1.00, 1.41)
*Marital Status*	
Married/partnered	Reference
unmarried/partnered	1.12 (0.91, 1.37)
*Education Level*	
College/Adv degree	Reference
Some college/Tech	1.13 (0.94, 1.35)
High school or less	0.74 (0.56, 0.98)*
*Income*	
<$35 k	Reference
$35 k to < $75 k	1.16 (0.95, 1.40)
$75 k and over	1.14 (0.90, 1.44)
*Previous Pregnancy*	
No	Reference
Yes	0.65 (0.53, 0.80)***

*p < 0.05, **p < 0.01, ***p < 0.001

^*a*^Dichotomized variable (4 vs. 1–3)

### Influence of contextual framing on attitudes about DMD NBS

As shown in Table 3, there were framing effects on DMD NBS attitudes. Participants who read about DMD NBS without the context of mandatory NBS (isolation group) reported DMD NBS to be less important and reported that they would worry more about DMD NBS results than those who saw either a bundled or unbundled panel (F = 3.40, *p* < 0.05; F = 3.48, *p* < 0.05, respectively). Participants’ social normative beliefs (i.e., reporting how they think what most other people believe) were associated with bundling; those who viewed DMD NBS without the context of mandatory NBS reported significantly weaker social normative beliefs around the endorsement of both NBS in general and DMD NBS specifically than those who also viewed mandatory NBS (in either the bundled or unbundled format) (F = 20.01, *p* < 0.01; F = 9.29, *p* < 0.01, respectively).

**Table 3:** Attitudes about Duchenne muscular dystrophy (DMD) newborn screening (NBS)

	Mandatory NBS Context			
Attitude Measure	*Bundled*	*Unbundled*	*No Context*	
	Mean (SD)	Mean (SD)	Mean (SD)	F
Subjective norms around NBS in general^*a*^	5.64 (1.52)	5.71 (1.49)	5.30 (1.63)	20.01**
Subjective norms around DMD NBS^*a*^	5.53 (1.57)	5.60 (1.54)	5.31 (1.56)	9.29**
How important is it to test for DMD?^*b*^	4.08 (1.09)	4.12 (1.00)	4.00 (1.05)	3.40*
How important is it to see the results?^*b*^	4.42 (0.98)	4.46 (0.90)	4.38 (1.01)	1.80
How important is it to share the results?^*b*^	2.68 (1.34)	2.62 (1.31)	2.61 (1.35)	0.74
How much would you worry about results^*c*^	3.42 (1.25)	3.45 (1.20)	3.56 (1.20)	3.48*
Results may help me prepare for the future^*d*^	4.39 (0.83)	4.41 (0.79)	4.39 (0.77)	0.21
Results may affect if I have more children^*d*^	3.21 (1.22)	3.19 (1.19)	3.20 (1.21)	0.08
Results may affect how I treat my child^*d*^	2.89 (1.35)	2.93 (1.34)	2.92 (1.35)	0.24
Others would treat my child differently^*d*^	3.58 (1.06)	3.64 (0.96)	3.66 (1.02)	1.73

*p < 0.05, **p < 0.01

^*a*^7-point Likert scale: 1 = Most people agree that it is important for babies to be tested for as many genetic diseases as possible/with getting the DMD test?; 7 = Most people disagree that it is important for babies to be tested for as many genetic diseases as possible/with getting the DMD test?

^*b*^5-point Likert scale: 1 = Not at all important; 5 = Very important

^*c*^5-point Likert scale: 1 = Not at all; 5 = Very much

^*d*^5-point Likert scale: 1 = Strongly disagree; 5 = Strongly agree

## Discussion

Parents are increasingly faced with the decision whether or not to participate in optional NBS as technology advances. Questions about how best to present such testing will become more important as NBS programs expand. This is the first study to look at the influence of contextual framing of optional NBS information on intended participation. Our results suggest that when people were not given the context of broader mandatory NBS, they were more hesitant to choose optional NBS testing and found it less appealing overall.

Recent work has identified factors of information presentation and choice that influence NBS decision making, including the routinization of procedure [22] and distinct optional NBS invitations [24]. Our study suggests that framing within NBS programs is another influence. It is possible that reading about mandatory NBS as a context for DMD NBS reminded participants about the benefits of NBS. Those participants were primed to consider the value of optional DMD NBS and evaluated the benefits of optional NBS differently. It is also possible that after viewing the number of mandatory NBS tests (either as “50” or by test), participants saw DMD NBS as just another test to add to the list.

Participants reading about DMD NBS in the context of mandatory NBS (either bundled or unbundled) reported the test to be more important and that they would worry less about the results. This is consistent with previous research finding parents without general NBS information had higher levels of worry after their infants' abnormal cystic fibrosis NBS, compared to parents with basic NBS information [29]. They also had stronger subjective normative beliefs around the endorsement of both NBS in general and the optional NBS test specifically, which have previously been shown to influence actual NBS decisions [27]. Taken together, our results suggest that the existence of accompanying mandatory NBS information, whether bundled or not, seemed to be very relevant to participants’ interest in optional NBS tests. Additionally, our patient characteristic findings that having a previous pregnancy and identifying as African American made one less likely to choose DMD NBS mirrors previous research, which showed significant effects of experiential knowledge in NBS decision making [17, 23] and African American race in optional NBS [30].

Our findings are tempered by a few limitations. First, our use of Internet-administered studies using hypothetical scenarios may not have evoked the same feelings or decision-making processes that would be present in a true population of DMD NBS decision makers, actual parents. However, our study materials mirrored those used in real-world contexts, especially materials used at the Columbus Children’s Research Institute for the Statewide Newborn Screening for Duchenne Muscular Dystrophy [19]. Second, our subject pool was also comprised of people who voluntarily chose to take surveys, which may limit generalizability. Finally, by virtue of the study design and study population, DMD NBS decision-making was conceptualized as a solo process and not one done with clinicians, partners and/or family members. An ideal scenario is a well-informed decision that includes at least two people [31]; in such a case the presence of additional decision makers may alter the impact of how DMD NBS information is communicated. However, as previous research has shown, NBS decisions are rarely an ideal process, with inconsistent information and incomplete discussions [9, 32]. This is particularly true for pilot NBS studies [33]. Thus, our study may actually resemble a true optional NBS decision: one made quickly with little advance discussion or education.

Technological innovation has created a “therapeutic gap”, in which one can screen for a disease before an effective treatment exists. With increasing advocacy [34] and technology, the NBS therapeutic gap is likely to continue expanding. Therefore it is important to consider the possible influences on decision makers before policies and universal guidelines are set regarding the communication of optional NBS information. Our findings demonstrate the possibility of greater or lesser test uptake simply due to structural differences in communications and program design, optional NBS managed as a separate entity or as an add-on to either a single, bundled package of mandatory NBS or as a panel of separate mandatory NBS tests. This is an important finding for clinicians to take note of; the context in which NBS tests are presented has the power to affect parental attitudes and decisions.

## Conclusions

With clinical trials testing emerging DMD treatments [35, 36], DMD NBS has come to the forefront of discussions about the inclusion of optional NBS in mandatory NBS [8, 37]. Our results suggest that viewing optional NBS information without the larger context of mandatory NBS impacts decision making. Several studies have explored the challenges in NBS education [9, 38–40]. However, evidence-based studies with parents considering optional NBS are needed to determine the relative importance of various communication factors in driving parents’ decisions. The effect of context seemed important regardless of how much detail is provided regarding mandatory NBS. Further research to improve our understanding of the significant influences on parental decisions regarding optional NBS decision making is required to inform future optional NBS practices and to help guide clinical and health communication practice as well as health policy.

## Additional file

Additional file 1:
**The three study brochures viewed by participants in each condition: 1) bundled 2) unbundled 3) isolation.**

## Abbreviations

NBS: Newborn screening
DMD: Duchenne muscular dystrophy
MTurk: Mechanical Turk
