# Supplementary material for: Framing optional genetic testing in the context of mandatory newborn screening tests
Source: BMC Med Inform Decis Mak. 2015 Jun 27;15:50. doi: 10.1186/s12911-015-0173-3 (PMC4485334; doi:10.1186/s12911-015-0173-3)
Supplement: Additional file 1: — The three study brochures viewed by participants in each condition: 1) bundled 2) unbundled 3) isolation. [file 12911_2015_173_MOESM1_ESM.pdf]

Do I Want My Newborn Son Screened for Duchenne Muscular Dystrophy?

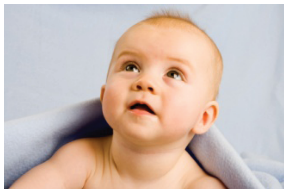

What is Duchenne (Doo-shen) muscular dystrophy (DMD)?

- DMD is a genetic condition that causes all muscles in the body to become weak over time.
- Clear signs of DMD are not present at birth, and usually don't appear until age 2-3.
- Boys with DMD might walk later than other boys and eventually boys with DMD need a wheelchair.
- Boys with DMD have a shortened lifespan.
- Treatment can slow down the disease, but there is *no* cure.

Why might I want my son screened for DMD?

- Without screening it might take years before DMD is diagnosed.
- Services like physical therapy can be started early.
- Treatment can slow down the progress of DMD.
- Knowing if your son has DMD can help you plan for the future.

Why might I *not* want my son screened for DMD?

- Although treatments are available, there is *no* cure.
- An early diagnosis could change how you treat your son.
- You might want to think your son is healthy until symptoms begin.

How is DMD screening done?

- *All* babies have a few drops of blood taken from their heel after birth.
- This blood is used to test for 50 genetic diseases that can be treated early to prevent serious problems or death.
- This test is called Newborn Screening (NBS).
- The *same* blood sample can be used to screen for DMD. You will not have to come to the hospital at an additional time or draw more blood.

Do I Want My Newborn Son Screened for Duchenne Muscular Dystrophy?

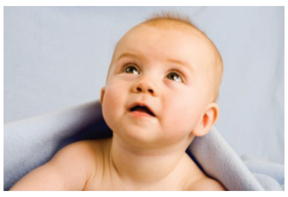

What is Duchenne (Doo-shen) muscular dystrophy (DMD)?

- DMD is a genetic condition that causes all muscles in the body to become weak over time.
- Clear signs of DMD are not present at birth, and usually don't appear until age 2-3.
- Boys with DMD might walk later than other boys and eventually boys with DMD need a wheelchair.
- Boys with DMD have a shortened lifespan.
- Treatment can slow down the disease, but there is *no* cure.

Why might I want my son screened for DMD?

- Without screening it might take years before DMD is diagnosed.
- Services like physical therapy can be started early.
- Treatment can slow down the progress of DMD.
- Knowing if your son has DMD can help you plan for the future.

Why might I *not* want my son screened for DMD?

- Although treatments are available, there is *no* cure.
- An early diagnosis could change how you treat your son.
- You might want to think your son is healthy until symptoms begin.

How is DMD screening done?

- *All* babies have a few drops of blood taken from their heel after birth.
- This blood is used to test for 50 genetic diseases that can be treated early to prevent serious problems or death.
- This test is called Newborn Screening (NBS).
- The *same* blood sample can be used to screen for DMD. You will not have to come to the hospital at an additional time or draw more blood.

Newborn Screening Tests

Amino Acid Disorders

1. Argininemia
2. Argininosuccinic acidemia
3. Citrullinemia Type I, II
4. Homocystinuria
5. Hypermethioninemia
6. Maple syrup urine disease (MSUD)
7. Phenylketonuria (PKU)
8. Benign hyperphenylalaninemia defect
9. Biopterin cofactor biosynthesis defect
10. Biopterin cofactor regeneration defect
11. Tyrosinemia Type I, II, III

Fatty Acid Oxidation Disorders

12. Carnitine acylcarnitine translocase deficiency
13. Carnitine palmitoyl transferase I deficiency
14. Carnitine palmitoyl transferase II deficiency
15. Carnitine uptake defect
16. Dienoyl-CoA reductase deficiency
17. Glutaric acidemia type II
18. Long-chain L-3-hydroxy acyl-CoA dehydrogenase deficiency
19. Medium/short-chain L-3-hydroxy acyl-CoA dehydrogenase deficiency
20. Medium-chain acyl-CoA dehydrogenase deficiency
21. Medium-chain ketoacyl-CoAthiolase deficiency
22. Short-chain acyl-CoA dehydrogenase deficiency
23. Trifunctional protein deficiency
24. Very long-chain acyl-CoA dehydrogenase deficiency

Hemoglobinopathies

25. S/Beta thalassemia
26. S/C disease
27. Sickle cell anemia
28. Variant hemoglobinopathies
29. Hemoglobin H disease

Organic Acid Disorders

30. 2-Methyl-3-hydroxy butyric aciduria
31. 2- Methylbutyryrl-CoA dehydrogenase deficiency
32. 3-Hydroxy 3-methylglutaric aciduria
33. 3-Methylcrotonyl-CoA carboxylase deficiency
34. 3-Methylglutaconic aciduria
35. Beta-ketothiolase deficiency
36. Glutaric acidemia type I
37. Isobutyryl-CoA dehydrogenase deficiency
38. Isovaleric acidemia
39. Methylmalonic acidemia cobalamin disorders (Cbl A,B)
40. Methylmalonic aciduria with homocystinuria (Cbl C,D)
41. Methylmalonic acidemia methylmalonyl-CoA mutase
42. Multiple carboxylase deficiency
43. Propionic acidemia

Endocrine Disorder

44. Congenital adrenal hyperplasia (CAH)
45. Congenital hypothyroidism (CH)

Other Disorders

46. Biotinidase deficiency
47. Galactosemia (GAL)
48. Cystic Fibrosis (CF)
49. T-cell related lymphocyte deficiencies
50. Severe combined immunodeficiency (SCID)

Optional Tests

Duchenne muscular dystrophy (DMD)

Do I Want My Newborn Son Screened for Duchenne Muscular Dystrophy?

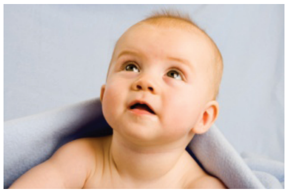

What is Duchenne (Doo-shen) muscular dystrophy (DMD)?

- DMD is a genetic condition that causes all muscles in the body to become weak over time.
- Clear signs of DMD are not present at birth, and usually don't appear until age 2-3.
- Boys with DMD might walk later than other boys and eventually boys with DMD need a wheelchair.
- Boys with DMD have a shortened lifespan.
- Treatment can slow down the disease, but there is *no* cure.

Why might I want my son screened for DMD?

- Without screening it might take years before DMD is diagnosed.
- Services like physical therapy can be started early.
- Treatment can slow down the progress of DMD.
- Knowing if your son has DMD can help you plan for the future.

Why might I *not* want my son screened for DMD?

- Although treatments are available, there is *no* cure.
- An early diagnosis could change how you treat your son.
- You might want to think your son is healthy until symptoms begin.

How is DMD screening done?

- DMD screening will not require you to come to the hospital at an additional time.
